# Early Diagnosis of Hemophagocytic Lymphohistiocytosis in an Adult Presenting With Sickle Cell Disease: A Case Report

**DOI:** 10.7759/cureus.64411

**Published:** 2024-07-12

**Authors:** Andres E Prieto-Torres, Andres F Rojas-Torres, German I Salguedo, Humberto Martinez-Cordero

**Affiliations:** 1 Internal Medicine, Hospital Militar Central, Bogotá, COL; 2 Hematology and Oncology, Hospital Militar Central, Bogotá, COL; 3 Hematology, Hospital Militar Central, Bogotá, COL

**Keywords:** immunoglobulin, dexamethasone, hemoglobin s, hemophagocytic lymphohistiocytosis, sickle cell disease

## Abstract

Hemophagocytic lymphohistiocytosis (HLH) is a rare but potentially life-threatening syndrome characterized by excessive immune activation and tissue inflammation. This case report describes the early diagnosis of HLH in an adult patient who initially presented with a febrile syndrome associated with low back pain. The patient, a 33-year-old male, exhibited bicytopenia, hepatosplenomegaly, and hyperferritinemia without a previous diagnosis of sickle cell disease (SCD). Diagnostic challenges arose due to the overlapping clinical manifestations of SCD and HLH and their uncommon association. However, timely recognition and intervention were achieved through comprehensive diagnostic evaluations, including a bone marrow biopsy. The patient was promptly started on an appropriate therapeutic regimen, which led to significant clinical improvement. This case underscores the importance of considering HLH in the differential diagnosis of adults presenting with hematologic abnormalities and systemic inflammation. Early diagnosis and treatment are critical to improving outcomes for patients with this complex and severe disorder.

## Introduction

Sickle cell disease (SCD) is a monogenetic disorder caused by a homozygous mutation in the beta-globin gene, resulting in the replacement of glutamic acid with valine at the sixth amino acid position, producing sickle hemoglobin (HbS) [1]. The life expectancy of patients with this disease is, on average, 20 years shorter than that of the general population [2]. Approximately 300,000 children are born annually with the severe homozygous form, the vast majority in sub-Saharan Africa and India [3].

The pathophysiology of HbS is characterized by the polymerization of hemoglobin when deoxygenated in capillary beds, causing a distortion of its normal structure and acquiring the characteristic sickle shape. This increases adhesion molecules, facilitating their binding to the endothelium and platelets, leading to occlusive processes responsible for ischemia (acute pain, acute chest syndrome, and avascular necrosis) with secondary endovascular inflammation. Additionally, sickle cells hemolyze rapidly, generating a compensatory increase in reticulocytes and secondary endothelial dysfunction associated with infarction phenomena, pulmonary hypertension, priapism, and leg ulcers, among others. Patients with a heterozygous genotype tend to present complications such as splenic infarcts and acute pain episodes later in life [4].

On the other hand, hemophagocytic lymphohistiocytosis (HLH) is a disorder in which patients often suffer from recurrent fever, cytopenias, liver dysfunction, and a sepsis-like syndrome that rapidly progresses to multiorgan failure. It is generally a hyperinflammatory hyperferritinemic syndrome triggered by T-lymphocytes with the potential to result in a fatal cytokine storm [5]. It can be primary in cases of inherited disorders with a Mendelian pattern or secondary, mostly due to infectious processes, especially viral etiology. In adults, neoplasms, particularly lymphomas, are a common cause [6].

The diagnosis of this syndrome uses the HLH-2004 criteria, where individuals require five or more of the eight diagnostic criteria: fever, splenomegaly, cytopenias (hemoglobin less than 9 g/dL, platelets less than 100 x 109/L, and neutrophils less than 1000/L), hypertriglyceridemia and/or hypofibrinogenemia (fasting triglycerides greater than or equal to 265 mg/dL, fibrinogen less than 150 mg/dL), hemophagocytosis in bone marrow, spleen, or lymph nodes, low or absent NK cell activity, ferritin greater than or equal to 500 mcg/L, and sCD25 greater than or equal to 2400 U/mL (treatment is indicated even if the minimum criteria are not met but suspicion is high) [7].

Hyperferritinemia will always be an inclusion criterion for considering the possibility of HLH. Ferritin levels in adults are usually greater than 7000 to 10000 mcg/L and rarely exceed 100000 mcg/L. As a marker, it does not have the same diagnostic performance as in children, and there is no universal consensus to define its diagnostic performance in adults [8]. Other characteristics such as hyperbilirubinemia, hepatomegaly, elevated transaminases, elevated lactate dehydrogenase, and D-dimer levels support the diagnosis without being part of the original criteria and are used to assess response to therapy [7].

The association between SCD and HLH is not widely elucidated, with isolated case reports, especially in children in Egypt and India, being much rarer in adults. We present the case of an adult presenting with SCD and developing secondary HLH.

## Case presentation

A 33-year-old black male patient from Puerto Asis, Putumayo, Colombia, presented to the emergency department with a 13-day history of lumbar pain associated with myalgias and intermittent fever. The patient had a diagnosis of lumbar spinal stenosis with right L5 root compression and distal herniation at L4-L5 and L5-S1, for which he declined surgical intervention. On physical examination at admission, the patient exhibited jaundice and right posterior cervical lymphadenopathy. Initial blood tests revealed the presence of normoblasts in peripheral blood (8 per 100 cells) and immature granulocytes (8% myelocytes and 3% metamyelocytes), indicating a leukoerythroblastic reaction. Elevated liver transaminases and bilirubin with a direct pattern were also noted (Table 1).

**Table 1:** Laboratory parameters from the present case MCV: mean corpuscular volume, MCH: mean corpuscular hemoglobin, RDW: red cell distribution width, AST: aspartate aminotransferase, ALT: alanine aminotransferase, LDH: lactate dehydrogenase, ND: no data

Test (unit)	April 7	April 8	April 10	April 25	Normal values
Leukocytes (x 10^9^/L)	24.93	19	10.14	7.56	4.5-11.3
Neutrophils (x 10^9^/L)	14.53	8.34	5.49	5.48	2.2-8.4
Lymphocytes (x 10^9^/L)	4.43	5.76	2.97	1.17	0.9-4.5
Platelets (x 10^9^/L)	103	54	37	101	150-450
Hemoglobin (g/dL)	12.6	9.1	5	10.2	12.1-16.6
Hematocrit (%)	32	23.7	12	30.6	35-49
MCV (fl)	72	72	69	91	80-100
MCH (pg)	27	27	27	30	26-34
RDW (%)	15.8	17	17	16	11.5-14.5
AST (U/L)	291	ND	ND	35	0-40
ALT (U/L)	429	ND	ND	59	0-41
Total bilirubin (mg/dl)	4.72	ND	3.75	0.58	0-1
Direct bilirubin (mg/dl)	2.94	ND	2.46	0.31	0-0.3
Alkaline phosphatase (U/L)	969	ND	ND	ND	40-129
LDH (U/L)	ND	ND	11149	1452	135-225
Creatinine (mg/dl)	1.17	ND	2.22	1.06	0.6-1.1
Urea nitrogen (mg/dl)	27	ND	76	16	8-23

The initial clinical approach focused on investigating infectious etiologies that could explain the hematological changes, acute liver injury, and renal damage. Tests for leptospirosis, hepatitis B, hepatitis C, toxoplasmosis, malaria, cytomegalovirus (CMV), and Epstein-Barr virus (EBV) were all negative.

Within the first 72 hours of hospitalization, the patient developed rapid and severe anemia, accompanied by progressive deterioration of consciousness, necessitating transfer to the intensive care unit for multiple red blood cell transfusions and ventilatory support. Further studies revealed peripheral blood smears with schistocytes and sickle cells, along with increasing normoblasts (42 per 100 cells). Levels of vitamin B12, folic acid, and thyroid-stimulating hormone were normal, but markedly elevated ferritin levels (92,093 ng/dL) and acute kidney injury were evident. The rapid onset of anemia and peripheral blood findings suggested a sickle cell crisis with secondary HLH (triglycerides 212 mg/dL; fibrinogen 421 mg/dL). Therefore, treatment with immunoglobulins (1.6 mg/kg split in two doses) and corticosteroids (dexamethasone 10 mg/m² for two weeks) following the adjusted HLH-94 protocol was initiated.

**Figure 1:**
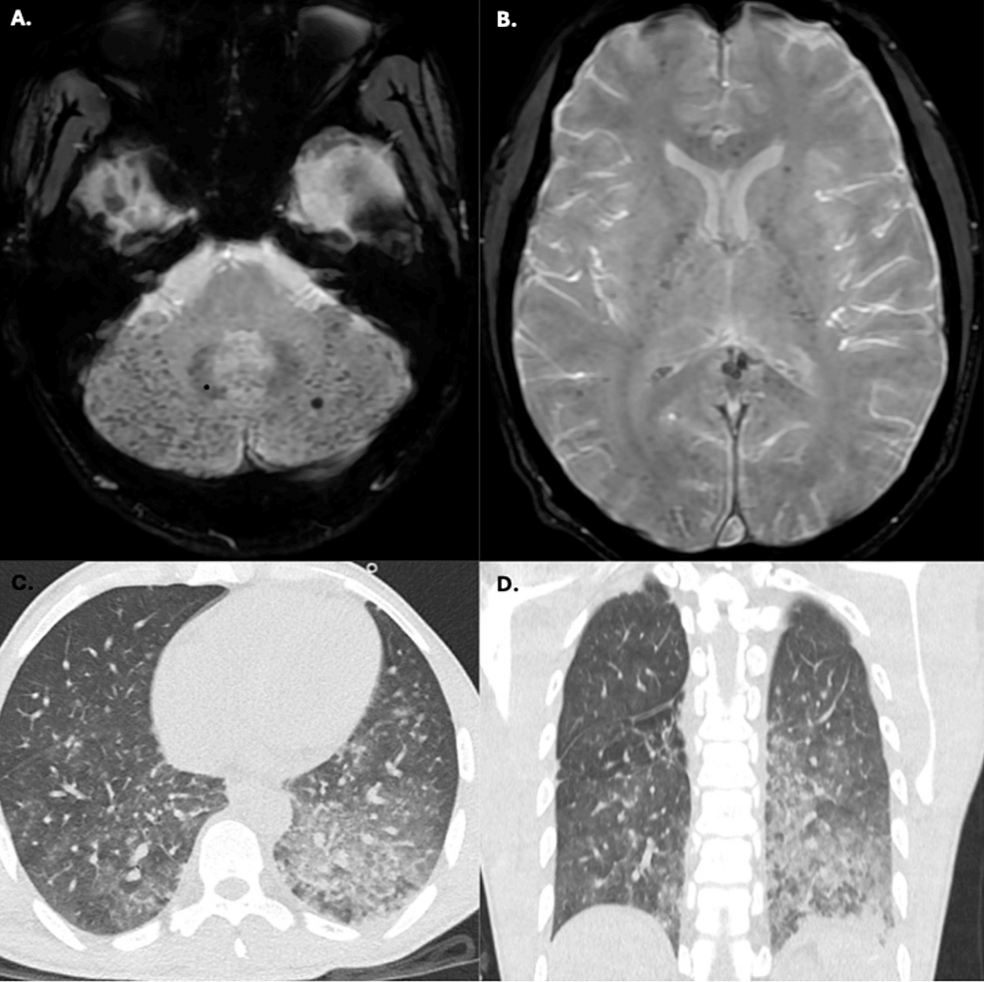
MRI and plain chest CT A-B: Countless microhemorrhages, predominantly subcortical, diffusely involving the cerebellum and, to a lesser extent, the cerebral parenchyma, the corpus callosum, and the corticospinal tracts. C-D: Alveolar opacities at the posterior basal segment of the left lung. Ground-glass opacities in both lower lobes. Numerous nodules and micronodules with a centrilobular distribution. MRI: magnetic resonance imaging, CT: computed tomography

As part of the cognitive deterioration assessment, a lumbar puncture revealed normal cerebrospinal fluid, and a brain MRI showed extensive microhemorrhagic involvement predominantly in the infratentorial region (Figure 1A-1B). Additionally, chest imaging indicated multilobar consolidative changes, leading to a diagnosis of acute chest syndrome associated with pneumonia (Figure 1C-1D). Abdominal ultrasound revealed hepatomegaly and a diffuse alteration in the echogenicity of the spleen. Given the possibility of functional asplenia, empirical antimicrobial therapy against encapsulated organisms was initiated using third-generation cephalosporins plus vancomycin.

Concurrent studies for the anemic syndrome showed a negative osmotic fragility test and hemoglobin electrophoresis in an acidic medium with a protein band in the migration zone of hemoglobin A and S (HbA 74%; HbS 11%; HbA2 14%), which was considered inconsistent with the clinical suspicion due to recent transfusion requirements.

Following the hematology service's treatment regimen, the patient exhibited significant clinical and paraclinical improvement (ferritin levels after 24 and 120 hours of immunoglobulin and corticosteroid treatment: 38,103 and 10,932 ng/dL, respectively). Early extubation was achieved, with progressive improvement in hemoglobin levels and general condition. Additionally, vaso-occlusive crises with cerebral ischemic lesions led to secondary panhypopituitarism, necessitating hormone replacement therapy and desmopressin for associated diabetes insipidus.

**Figure 2:**
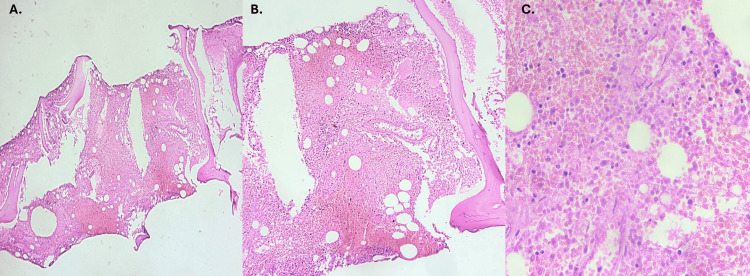
Histopathology images of the bone marrow biopsy A. 4x; B. 10x; C. 40x. Trabecular bone with hematopoietic intertrabecular tissue showing frequent foci of coagulative necrosis, interstitial hemorrhage, and some fibrin deposits. Coagulative necrosis changes in the bone marrow are generally associated with systemic events that compromise blood supply and have been described in vaso-occlusive crises due to sickle cell anemia.

During the diagnostic process, three bone marrow biopsies were performed. The first two were non-diagnostic due to massive hemolysis of the samples, while the third revealed coagulative necrosis in the bone marrow generally associated with peripheral vaso-occlusive crises due to sickle cell anemia (Figure 2). After 15 days of initiating the HLH-94 treatment regimen, the patient achieved complete clinical improvement and was discharged with a gradual tapering of dexamethasone, hormone replacement therapy, and hydroxyurea at a dose of 500 mg every 12 hours.

## Discussion

HLH was first described in 1939 in a case associated with a viral infection. Evidence of a familial predisposition to the development of the pathology was described in 1991. It is a condition with high mortality, with etiology primarily described in children secondary to viral infections (EBV and CMV), although there are increasing reports of less common causes such as dengue virus infections, bacterial infections, especially by *Mycobacterium tuberculosis*, and parasitic infections. Additionally, there is a rising number of cases associated with hematologic neoplasms, solid tumors, autoimmune diseases, and medications in adults [7]. While cases associated with sickle cell crises are not common, they have been described but are not easily recognizable, leading to unclear incidence rates. The clinical and laboratory characteristics of vaso-occlusive crises and HLH partially overlap, making their diagnosis challenging.

Upon reviewing the literature, isolated case reports have linked vaso-occlusive crises in patients with SCD to HLH. In the first case, a 35-year-old woman with a family history of sickle cell anemia and chronic ulcers on the lower limbs presented with painful crises in the limbs and a history of 23 units of transfused red blood cells with a baseline ferritin level of 442 ng/ml. She developed a recurrent fever unresponsive to antibiotics, negative etiological studies, progressive deterioration leading to transfer to the ICU, kidney injury requiring dialysis, cytopenias, and diffuse lymphadenopathies. Transient mild viremia for EBV was detected in extension studies, along with hyperferritinemia, hypertriglyceridemia, increased IL-2R, and hypofibrinogenemia. A bone marrow biopsy revealed hemophagocytosis. The patient improved with a regimen of dexamethasone and immunoglobulins. The second case involved a 56-year-old patient with HbSC disease presenting vaso-occlusive crisis and fever, with a transfusion history of 82 units of red blood cells and a baseline ferritin level of 199 ng/ml. Studies showed mild CMV viremia, progressive consciousness deterioration, increased ferritin and soluble IL-2R levels, and a biopsy confirming histiocytosis with hemophagocytosis. The patient rapidly improved with dexamethasone and etoposide [9].

A third case described a five-year-old child in Egypt, born to consanguineous parents, who presented with respiratory symptoms and pallor associated with severe anemia requiring red blood cell transfusion. Despite antibiotic treatment, the child developed fever, hepatosplenomegaly, normal volume anemia with leukocytosis and neutrophilia, elevated acute phase reactants, and a lung scan revealing a pulmonary abscess. Carbapenems were effective initially, but after three weeks, there was deterioration with fever, cytopenias, jaundice, and splenomegaly associated with hyperferritinemia and hypertriglyceridemia. A bone marrow biopsy showed normocellular marrow with increased erythropoiesis but no hemophagocytosis. The patient responded well to dexamethasone therapy [10]. The fourth case involved a 23-year-old patient with lumbar and thoracic pain, a fever, a history of occasional alcohol and cocaine use, and no transfusion history. On admission, the patient had hepatosplenomegaly, elevated lactate dehydrogenase, thrombocytopenia, and high ferritin levels. Protein electrophoresis revealed an HbSB phenotype, and a bone marrow biopsy revealed pronounced histiocytosis with excessive hemophagocytosis. Empirical treatment with ceftriaxone alone resulted in the patient's improvement [11]. Additional cases have been documented in pediatric SCD populations with HLH events triggered by coinfections of the hepatitis A-E virus, in adults with SCD developing HLH secondary to transfusions, and even associated with infections by *Escherichia coli*, *Proteus*, and mycobacteria [12-14].

Vaso-occlusive crises and HLH can lead to multi-organ damage and be fatal. Since the treatment for both conditions is different, considering HLH in sickle cell crisis patients who do not respond to standard therapy can be a life-saving measure. In the specific case of our patient, without a known diagnosis of SCD or transfusion history, it suggests a likely scenario of a late presentation with a heterozygous phenotype of the disease. The specific trigger of the sickle cell crisis and subsequent development of HLH in our patient is not entirely clear. Common infectious etiologies were appropriately ruled out, as seen in other reviewed case reports where, despite documenting viremias, they were transient and did not explain the symptom development. Furthermore, in one report, the association between zinc deficiency in SCD and thalassemias with hypercytokinemia and impaired NK activity exacerbated during vaso-occlusive crises was suggested as a potential cause of HLH [15].

It is worth noting that our patient had been experiencing general symptoms and pain for 13 days without signs of anemia. Importantly, the patient had recently traveled by plane from a city at 230 meters above sea level to Bogotá, which is at 2600 meters above sea level. Decompensation was observed 72 hours after arrival, potentially linked to the triggering of sickle cell crises in such altitude changes [16,17].

Although distinguishing between the laboratory findings of both conditions can be challenging, changes in the blood count, particularly reticulocytosis due to stress, with macrophages playing a crucial role in the bone marrow and erythropoietic centers, are described. The accelerated turnover impacts monocyte migration to these sites and plays a role in enucleating immature red cell forms, leading to the release of normoblasts to the periphery and the production of cytokines that induce leukocyte reactions [18].

While no evidence of hemophagocytosis in the bone marrow was found, as this is not pathognomonic and has poor sensitivity, its absence does not rule out the diagnosis in a highly suspicious scenario like ours. Additionally, the first two biopsies were uninterpretable due to massive hemolysis, and the third biopsy was taken after initiating treatment with immunoglobulins and corticosteroids. This timing could partially explain the findings, which, on the contrary, are highly specific to systemic vaso-occlusive crises. The patient's electrophoresis was suggestive but not diagnostic of SCD due to transfusions received during hospitalization. Nonetheless, the diagnosis was supported by the blood smear and clinical presentation.

The final question remains whether the sickle cell crisis alone can trigger HLH, possibly in relation to altitude changes, or if there was an undiagnosed viral process as a likely trigger for the entire clinical picture.

## Conclusions

HLH presents a complex and often fatal syndrome that can complicate various underlying conditions, including SCD. The literature underscores the challenge of diagnosing HLH due to overlapping clinical and laboratory features with vaso-occlusive crises. This case report highlights the necessity of considering HLH in patients with SCD who exhibit atypical responses to standard treatments, emphasizing the importance of early recognition and appropriate intervention to improve outcomes. Our case suggests that sickle cell crises, potentially exacerbated by environmental factors such as altitude changes, can precipitate HLH. Although diagnostic challenges remain, particularly in differentiating between these conditions, the interplay of stress-induced reticulocytosis and macrophage activity in bone marrow suggests a plausible link. Timely identification and treatment of HLH, especially in the context of sickle cell crises, are crucial for patient survival. Future research should focus on better understanding the triggers and pathophysiological mechanisms linking these conditions to enhance diagnostic accuracy and therapeutic strategies.
